# The standards of practice for delivery of polypharmacy and chronic disease medication reviews by general practice clinical pharmacists: a consensus study

**DOI:** 10.1007/s11096-022-01387-7

**Published:** 2022-03-23

**Authors:** Katie Earle-Payne, Paul Forsyth, Chris F. Johnson, Heather Harrison, Susan Robertson, Anita E. Weidmann

**Affiliations:** 1 NHS Greater Glasgow & Clyde, Renfrew Health and Social Care Centre, 10 Ferry Road, Renfrew, PA4 8RU United Kingdom; 2 School of Pharmacy and Life Sciences, Robert Gordon University, Sir Ian Wood Building, Robert Gordon University, Garthdee Road, Aberdeen, United Kingdom; 3 NHS Greater Glasgow & Clyde, Clarkston Court, 56 Busby Road, Glasgow, G76 7AT United Kingdom; 4 Faculty of Chemistry and Pharmacy, University Innsbruck, Innrain 80-82, Innrain 52c., 6020 Innsbruck, Austria

**Keywords:** Chronic disease medication reviews, Pharmacy practice, Polypharmacy reviews, Primary care, Standards of practice

## Abstract

*Background* General practice in the UK is experiencing a crisis. Greater multidisciplinary working is a potential solution. The new general practice contract in Scotland encourages this and includes a new pharmacotherapy service to be delivered by General Practice Clinical Pharmacists (GPCPs). Consensus is lacking for the standards of practice for delivery of pharmacotherapy medication reviews (which are polypharmacy and chronic medication reviews) as part of this service. *Aim* To identify and validate standards of practice for polypharmacy and chronic disease medication (pharmacotherapy level 3) reviews conducted by GPCPs. *Method* A two-phased mixed-methods consensus methodology was used. Phase 1: An expert group of GPCPs (n = 4) and clinical pharmacist managers (n = 2) responsible for delivering the pharmacotherapy service used a Modified Nominal Group Technique to generate potential standards. Phase 2: Two-round Delphi survey involving GPCPs with ≥ 1 year of experience of working in general practice (n = 159). *Results* The expert group identified 44 potential standards of practice for polypharmacy and chronic disease reviews. Practicing GPCPs indicated during the Delphi phase that the 44 standards were applicable to practice. The standards of practice covered seven main categories: skills, environment, qualifications, qualities and behaviours, knowledge, process and experience. *Conclusion* Practicing GPCPs indicated that the standards identified by the expert group are acceptable and valid for current practice and the delivery of polypharmacy and chronic medication reviews. The application of these standards to practice may help GPCPs and general practices to ensure equitable delivery of patient care.

**Supplementary Information:**

The online version contains supplementary material available at 10.1007/s11096-022-01387-7.

## Impacts on practice

- This study identified and validated standards of practice for general practice clinical pharmacists performing polypharmacy and chronic disease medication reviews.
- The standards covered seven main categories: skills, environment, qualifications, qualities and behaviours, knowledge, process and experience which were applicable to current practice as indicated by practicing general practice clinical pharmacists.
- Stakeholders may find these standards of use as a platform for further developing and aligning services and professional standards.

## Introduction

Healthcare provision around the world is evolving and globally there is a shift in pharmacy multidisciplinary working across Europe, North America and Australasia. There is a greater emphasis on drawing on pharmacists’ knowledge and skills as expert in medicines to optimise care and outcomes for people with polypharmacy and co-morbidities [1–4]. Pharmacists co-located in general practice clinics, focusing on delivering a range of interventions for patients receiving chronic medications, is becoming more common in many countries [5].

The recruitment crisis of doctors in general practice in the United Kingdom (UK) is well documented [6–8]. In part this is due to an aging populations with complex needs, advances in medical care, intensifying workloads, demands and pressures that compromise patient-centred care [8–10]. All of this, is driving GPs away from the National Health Service (NHS), their professions, and patients [9, 11]. There is a need to redress these pressures; to enable a better work–life balance to allow GPs to fulfil personal responsibilities and needs [11, 12].

In the UK pharmacists have worked alongside GPs in general practices for over 20 years to improve patient care, outcomes and, more recently, free GP time and capacity [5, 12–17]. Consequently, there is a drive to recruit more pharmacists to general practice and help general practice to continue to deliver patient services [18, 19]. The demand for General Practice Clinical Pharmacists (GPCPs) in Scotland has become more urgent since the introduction of the new General Medical Services (GMS) contract in Scotland in 2018 [19]. This contract includes a new three-tiered pharmacotherapy service to be delivered by GPCPs with the aim to optimise patient care and free up GP capacity. Level 1—‘core’ service—includes authorising all prescription requests, immediate discharge letters, outpatient letters, medicine safety reviews and monitoring high risk medicines. Level 2—‘additional advanced’ service- includes medication reviews of ≥ 5 medicines and resolving high risk medicine problems with level 3—‘additional specialist’ service—including polypharmacy reviews and specialist clinics for chronic pain, heart failure, diabetes, etc. [19].

The GMS contract is clear on requirements to provide level 1 and 2 services, however, level 3 service necessitates definition as to how this will be provided to ensure uniformity of care. One of the ways this can be achieved is by developing a set of standards a pharmacist has to meet in order to perform these tasks. A standard is a statement that provides a broad framework and describes how safe and effective care is delivered, hence providing support for pharmacists to improve and shape services [19].

Several sets of standards for pharmacy professionals are available from national organisations within the UK, North America and Australasia. Internationally, in 2012 the International Pharmaceutical Federation (FIP) has published a Global Competency Framework for Pharmacists which provided the basis for national standards set by many pharmacy regulatory bodies around the globe [20–23]. In the UK, the General Pharmaceutical Council and Royal Pharmaceutical Society published standards for safe and effective pharmaceutical care [24–27], and the Centre for Postgraduate Pharmacy Education England and NHS Education for Scotland published standards for GPCPs and a GPCP competence framework [28, 29]. All these publications have common themes such as patient-centeredness, good communication, multidisciplinary working, professionalism and speaking up about concerns. While a range of specialist clinical pharmacy service standards and competencies have been defined within the UK and elsewhere, a Canadian study is the only one that considered and developed competencies for pharmacists working in general practice [30–38]. This study however did not define standards of practice for the delivery of medication reviews, and hence more research is needed in this area.

### Aim

To identify and validate standards of practice for polypharmacy and chronic disease medication reviews (pharmacotherapy level 3) conducted by GPCPs.

### Ethics approval

This study received ethical approval from Robert Gordon University School of Pharmacy and Life Science Research Committee, approval number # S225, on 20/12/19.

## Method

### Design

Two-phase consensus study. Phase 1 applied a Modified Nominal Group Technique (NGT) to generate and achieve consensus on standards of practice for polypharmacy or chronic disease medication review via an expert group. Phase 2, a two-round Delphi questionnaire was applied to gain broader GPCP workforce consensus and validate the standards, in line with CREDES (Conducting and Reporting of Delphi Studies) guidelines (Supplementary file1) [39].

### Setting

The UK NHS is a taxpayer funded healthcare system. NHS Greater Glasgow and Clyde (NHSGGC) health board provides healthcare services for a diverse population of 1.2 million people across a varied urban region containing 260 general practices. NHSGGC covers six Health and Social Care Partnerships (HSCPs) and in 2020 employed 159 GPCPs working in general practices.

#### Phase 1: modified NSGT

**Fig. 1:**
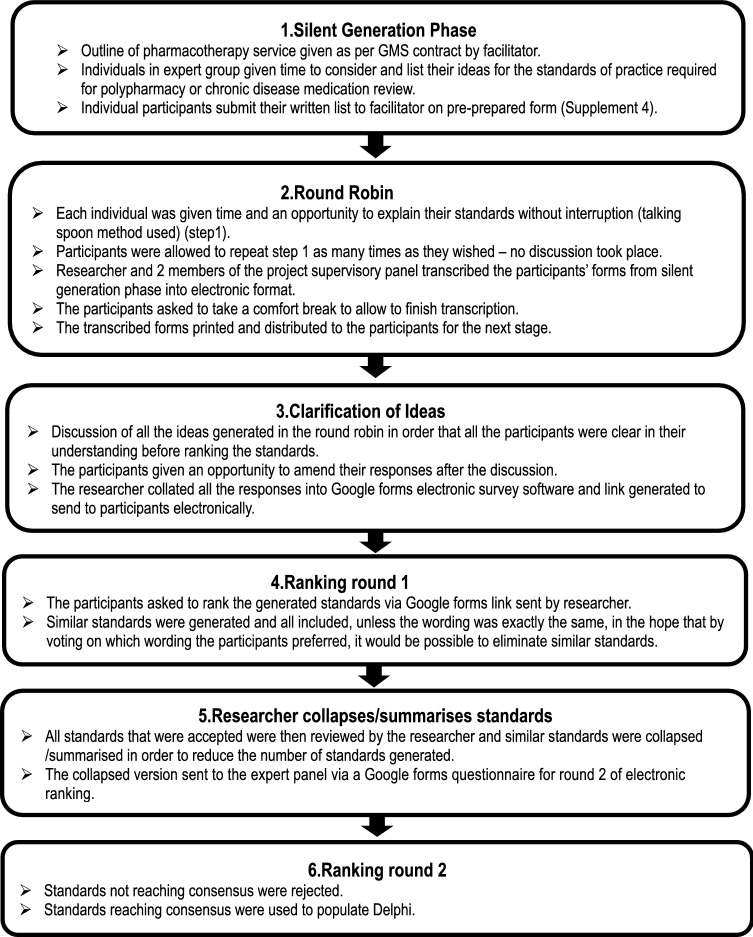
Modified Nominal Group Technique process

A modified NGT was used, see Fig. 1. An expert group was recruited from experienced GPCPs meeting the following inclusion criteria: experience running regular established clinics for ≥ 4 years and prescribing on a regular basis, and lead clinical pharmacists responsible for service delivery. All, were invited to participate. Co-authors were excluded from participation to reduce bias. A study recruitment email, was sent to all 159 GPCPs via HSCP lead clinical pharmacists in January 2020. A reminder email was sent to encourage participation 2 weeks later. Purposive sampling was employed to ensure that the panel was sufficiently diverse in their experience to ensure validity of results [40]. The NGT was facilitated by the authors (KEP, PF, CJ and HH).

*Data generation* Consensus methods such as NGT are frequently used for structured idea generation and achieving agreements in healthcare research, especially when little is known on the subject and the opinions of the experts in the field are sought with the benefit of equal participation [41, 42]. A traditional NGT is a structured face-to-face method of generating consensus involving four key stages—idea generation, round robin, clarification of ideas and voting/ranking [43, 44].

This study employed a modified NGT, applying electronic voting/ranking after face-to-face silent idea generation, round robin and clarification of ideas by the expert group (Fig. 1, Stage 1–3). Similar generated standards were then summarised into single standards between round 1 and 2 of ranking. A standardised data collection form for the silent idea generation phase was developed by the lead author (KEP) and the modified NGT process were piloted by KEP and two co-authors (PF and CJ).

*Data analysis* The standards generated by each participant during the face-to-face meeting populated an electronic Google forms questionnaire and was sent to each participant for ranking; involving two rounds of ranking (Fig. 1). A 5-point Likert-type scale (‘strongly agree’, ‘agree’, ‘neutral’, ‘disagree’ and ‘strongly disagree’) was used. Consensus was defined as ≥ 80% of participants agreeing (‘strongly agree’ or ‘agree’) for a standard to be accepted. Where participants selected ‘strongly disagree’ or ‘disagree’, the—standard was rejected. Standards achieving consensus in round 2 populated the Delphi phase.

#### Phase 2: Delphi

Delphi questionnaires allow for wide participation and anonymity with two rounds commonly used in the literature, because it is known that > 2 rounds can cause participant fatigue and dropout [44, 45], therefore a two-round Delphi was conducted. The first round was informed by the modified NGT compiled by KEP and piloted by PF and a practicing GPCP.

All experienced GPCPs (≥ 1 year experience in primary care) were invited to participate. This was to allow for a broad and relevant consensus to be achieved. Convenience sampling was employed. Co-authors were excluded from participation to reduce bias. A study recruitment email was sent to all 159 GPCPs via HSCP lead clinical pharmacists for round 1 (March 2020) and round 2 (September 2020—delayed due to COVID-19 pandemic). The email outlined the study, provided a link for the online Webropol questionnaire and requested that participants completed it within a 2 week timeframe. Participants consent to participate was sought prior to completing the questionnaire.

*Data analysis* The participants rated the presented standards for inclusion using a 5-point Likert scale (see above). Consensus setting varies between NGT and consensus studies, with most common ranges quoted between 70 and 90% [44–48]. Consensus was defined as ≥ 70% of agreement (agree/strongly agree—standard accepted) or disagreement (disagree/strongly disagree—standard rejected). Standards achieving consensus (≥ 70% agree or disagree) were removed before the second round. The results from the second round were analysed using the same method.

## Results

### Demographics

The NGT expert group consisted of four GPCPs and two lead clinical pharmacists. All participants were female; median age 42 (range 37–53) years old, with a median of 13 years (range 7–20) experience working in general practice. All had experience of running a range of regular clinics (polypharmacy, respiratory, pain, hypertension, cardiology, diabetes) 67% (n = 4) of whom currently delivered patient-facing clinic. All had additional postgraduate qualifications and were independent prescribers.

The Delphi phase captured responses from 59 (37%) and 86 (54%) GPCPs practicing in NHSGGC, for round 1 and 2 respectively (Table 1). Respondent characteristics were similar for round 1 and 2. They were of similar ages, the majority were female, had gained postgraduate qualifications and were prescribers. A similar proportion of GPCPs had experience in running clinics in both groups. In round 2 fewer clinicians reported that they currently ran medication review clinics.

**Table 1:** Delphi phase, respondent characteristics

Characteristics	Round 1 (n = 59)	Round 2 (n = 86)
Age, median (range) years	39 (26–60)	38 (25–63)
Gender, female, n (%)	51 (86)	77 (90)
*Postgraduate qualifications*		
Independent prescriber, n (%)^a^	53 (90)	71 (83)
Masters, n (%)	19 (32)	23 (27)
Clinical diploma, n (%)	13 (22)	25 (29)
Clinical certificate, n (%)	7(12)	10 (12)
Previous experience running clinics, n (%)	52 (88)	74 (86)
Current experience running clinics, n (%)	36 (61)	24 (28)
Experience in primary care, median (range) years	6 (1–22)	4 (1–23)

^a^Independent prescribing is an additional professional qualification that allows a pharmacist in the UK to prescribe

### Standards generation

#### Phase 1: modified NGT

The expert group initially generated 121 standards during the silent generation and round robin phases. Clarification of ideas and the first round of ranking rejected 25 standards: 13 due to duplication; 8 relating to medication review ‘time’; 4 relating to governance (Supplementary file 2). The remaining 96 standards were collapsed/summarised into 47 standards in seven categories (Fig. 1, Table 2) Of the 47 standards: 11 (23%) related to ‘Skills’, 9 (19%) to ‘Environment’, 7 (15%) ‘Qualifications’, 6 (13%) ‘Process’, 6 (13%) ‘Qualities and Behaviours’, 5 (11%) ‘Knowledge’ and 3 (6%) ‘Experience’. Ranking round 2 then resulted in three standards not reaching consensus and being rejected, two of which related to ‘Process’ and one to ‘Environment’. The 44 standards reaching consensus populated the Delphi phase.

**Table 2:** Standards for practice generated during NGT phase ranking round 2 (n = 6) and Delphi phase round 2 (n = 86) by category

Standards of practice	NGT	Delphi
*Skills*		
1. Demonstrates patient-centred approach (ability to involve patient in decisions relating to their care and ensure patient understands their care plan)	**●**	**●**
2. Demonstrates holistic view^1^ of patient	**●**	**●**
3. Demonstrates good listening skills	**●**	**●**
4. Demonstrated the ability to manage complex patients	**●**	**●**
5. Demonstrates an understanding that sometimes no change is appropriate at this point, ‘planting the seed’ to prepare for the future	**●**	**●**
6. Demonstrates ability to assess and balance risk of harm versus benefits of prescribing or de-prescribing	**●**	**●**
7. Demonstrates ability to effectively safety net^2^ when changes are made to medication	**●**	●*
8. Demonstrates understanding of where to signpost^3^ patients for non-pharmacological interventions, which are often just as important as pharmacological ones	**●**	**●**
9. Demonstrates motivational techniques beneficial for encouraging self-management and lifestyle change for most chronic disease areas	**●**	**●**
10. Demonstrates the ability to interpret test results relevant to conditions (e.g. ECG, spirometry, bloods)	**●**	**●**
11. Demonstrates good time management—ability to work within agreed time frames	**●**	**●**
*Environment*		
12. Has peer support—everyone has a mentor or appraisal additional to Knowledge Skills Framework	**●**	**●**
13. Has network of support people/experts to ask if you need advice including multidisciplinary support, peer support from other pharmacists and GPs	**●**	**●**
14. Participates in peer review in specialist areas. Discussing cases with a peer or another clinician with expertise in a particular clinical area to review competence and practice and ensure in-line with peers	**●**	**●**
15. Has mentor/advisor that could be called upon for advice	**●**	**●**
16. Has support from practices and buy in from other practice prescribers to ensure sustainability of prescribing services and changes made to patients’ medication	**●**	**●**
17. Has adequate time to allow full polypharmacy reviews to be conducted	**●**	**●**
18. Where necessary has flexibility for repeated appointments with patients	**●**	**●**
19. Has flexibility to conduct reviews in patients’ homes if appropriate i.e. the place most suitable for the patient	**●**	**●**
20. Has Royal Pharmaceutical Society membership is optional but may be advantageous as opens mentoring support and clinic information	**♦**	
*Qualifications*		
21. Qualified independent prescriber, with up-to-date knowledge and prescribing competence in the area in which they prescribe	**●**	**●**
22. Has relevant post graduate qualifications depending on individual career path (e.g. clinical or GPCP framework)	**●**	●*
23. Has completed consultation skills training (NES^1^ and video recording including feedback)		
24. Has completed NES clinical examinations course (and advanced if relevant to clinical area)	**●**	**●**
25. Has completed NES communication course	**●**	**●**
26. Has completed suicide prevention training	**●**	●*
27. Has completed behaviour skills training	**●**	●*
*Qualities and behaviours*		
28. Demonstrates self-awareness, self-motivated and the ability to work independently; understands own limitations and when (and where) to seek help	**●**	**●**
29. Demonstrates effective team working drawing on individual strengths	**●**	**●**
30. Takes responsibility for own actions	**●**	**●**
31. Demonstrates confidence to challenge issues appropriately (e.g. behaviours, prescribing, patient care etc.)	**●**	**●**
32. Demonstrates honesty	**●**	**●**
33. Demonstrates leadership (e.g. clinic development/patient care)	**●**	**●**
*Knowledge*		
34. Has understanding of role within a wider team and how the team functions as well as ability to work within individual GP practice structures and systems	**●**	**●**
35. Has knowledge of local and national formularies and guidelines	**●**	**●**
36. Has knowledge of new/progressing evidence	**●**	**●**
37. Has good understanding of resources to support clinical practice	**●**	**●**
38. Has understanding of brief interventions	**●**	**●**
*Process*		
39. Demonstrates evidence of quality improvement, via self—reflection/audit against specified standards. Intervals yearly or bi-yearly	**●**	**●**
40. Demonstrates evidence of reflection and continuous assessment—as an individual and with peers	**●**	**●**
41. Undertakes significant event analysis 1. NES: NHS Education for Scotland	**●**	**●**
42. Produces clear documentation throughout	**●**	**●**
43. Undertakes regular self-reflection of prescribing	**♦**	
44. Informs if unable to meet deadlines	**♦**	
*Experience*		
45. Has experience and utilises relevant clinical assessment and examination skills	**●**	**●**
46. Has experience in running clinics	**●**	●*
47. Has experience managing case load in therapeutic area	**●**	●*

*ECG* electrocardiogram; *GP* general practitioner

*Standards that did not achieving consensus Delphi round 1, but achieved consensus in round 2

**●** Consensus reached, ≥ 80% agreement in NGT and ≥ 70% in Delphi phases

**♦** Consensus not attained (NGT phase)

**_ __** New standards compared to the existing standards of practice for pharmacists found

_ _ Standards relevant to UK only and further clarification of these is given as supplementary file 3

^1^Holistic view: looking at overall health of patient including their physical psychological, social and spiritual wellbeing

^2^Safety**-**netting: information given to a patient or their carer during a primary care consultation, about actions to take if their condition fails to improve, changes or if they have further concerns about their health in the future

^3^Signposting: help patients understand, access and navigate services that improve their health

#### Phase 2: Delphi

The first round was completed by 59 (37%) GPCPs, and the second-round by 86 (54%). Consensus was reached during the second-round, all 44 standards proposed by the expert panel being accepted (Table 2).

‘Skills’ was the largest category with 11 standards. These focused on, but were not limited to, taking a holistic patient-centred view when carrying out level 3 reviews, as well as demonstrating the ability to manage complex patients and balance risk of harm and benefits when prescribing and deprescribing. There was also emphasis on signposting and non-pharmacological interventions, the ability to interpret test results for relevant conditions and good time management.

‘Environment’ was the next largest category (n = 8). These standards focused mainly on two areas, firstly, peer support and mentoring for all GPCPs from pharmacists and the wider multidisciplinary team. Secondly, a culture of support within practice ‘to allow full polypharmacy reviews to be conducted’ that incorporated flexibility for repeat appointments within practices and the capacity to conduct reviews in the setting that was ‘most suitable for patients’ e.g. the patients home.

The ‘Qualifications’ category (n = 7), indicated that the GPCPs performing level 3 reviews should be qualified independent prescribers, with up-to-date knowledge and be competent prescribers. Participants demonstrated consensus that GPCPs should have relevant postgraduate qualifications and undertake appropriate additional training such as consultation, communication and relevant clinical examination skills training, suicide prevention training and behaviour skills training.

‘Qualities and Behaviours’ standards (n = 6) focused on GPCPS demonstrating self-awareness, self-motivated and the ability to work independently, but were aware of their own limitations and sought help appropriately. Demonstrating good team working and drawing on individual multidisciplinary team members’ strengths and knowledge was also considered to be important.

‘Knowledge’ standards (n = 5) highlighted the importance of understanding the GPCP role within wider general practice and healthcare team, and the ability to work within different general practice structures and systems. A knowledge of local/national formularies, guidelines and resources to support clinical practice. However, ‘Process’ (n = 4) orientated standards focused on quality improvement, for the GPCP service and personal development through self-reflection and audit practice, and ensuring that good documentation was in place. Lastly, ‘Experience’ standards (n = 3) concentrated on relevant experience that the pharmacists should have in order to deliver effective and efficient service, such as GPCPs having relevant experience in clinical assessment and examination, running clinics and managing caseloads.

## Discussion

### Key findings

This study identified and validated 44 standards of practice specific to performing polypharmacy and chronic disease medication reviews in general practice. All 44 standards identified by the expert panel reached consensus and were accepted by the Delphi participants, who were experienced GPCPs that are expected to deliver these medication reviews.

The standards covered seven main categorises: skills, environment, qualifications, qualities and behaviours, knowledge, process and experience and concentrated on good communication and patient centeredness with ability to manage complex patients, leadership and team work as well as the ability to work independently. New standards, specific to complex polypharmacy and chronic disease medication reviews in general practice were also identified.

### Strengths

To the authors’ knowledge, this is the first study to identify and validate standards of practice and hence standardise polypharmacy and chronic disease medication reviews in general practice. Groups with a maximum of seven participants have been recommended for NGT [41]. The purposive sample of six participants in NGT was small enough to have a close face-to-face discussion, yet large enough to include a broad range of expertise. All the standards generated by the expert group (NGT) were accepted by the GPCPs (Delphi) which adds to the study’s validity. Participants’ demographics were comparable to the recent Scotland wide general practice workforce survey [14], however a higher proportion (83–90%) of respondents were prescribers, which may be explained by further encouragement for independent prescriber qualifications in recent years and the fact that more experienced pharmacists were asked to participate. Another strength was that this study was carried out within single regional health board pharmacy team that have similar management structures, formularies and clinical guidelines.

### Limitations

The response rate to Delphi of 37% (n = 59) (round 1) and 54% (n = 86) (round 2) may be considered low but is comparable to previous studies [34, 41]. The study was conducted during COVID pandemic which affected participation. A lower proportion of pharmacists reported current experience of running clinics in round 2 (32%) than round 1 (69%), due to routine clinic cancellations at the height of the pandemic and the second round being conducted during this period. Another potential limitation is that this study did not include non-pharmacy stakeholders i.e. GPs, practice nurses, policy makers, however future studies should consider including such key stakeholders. The NGT employed purposive sampling which resulted in an all-female panel and Delphi employed convenience sampling. These factors may have introduced potential biases, however, the NGT participants had worked in general practice for a median of 13 years, delivering regular clinics and the majority of their standards were accepted by practice GPCPs.

### Interpretation

There are a number of implications for policy and practice. The standards generated in our study were similar to those published by the FIP, American College of Clinical Pharmacy and in Canada and Australia, as well as locally in UK and Canadian studies where standards generated for pharmacists working in primary care focussed on patient care in relation to medication related needs [20–23, 38]. They listed patient-centeredness, effective communication, multidisciplinary collaboration, professionalism and up to date knowledge [21–24, 38].

Some of the standards identified, however, differ from the above publications, likely due to this study concentrating on identifying standards of practice specifically for conducting polypharmacy and chronic disease medication reviews within general practice. The ‘Qualifications’ and ‘Knowledge’ categories for standards necessitated pharmacists performing medication reviews to be a qualified prescribers, having completed specific clinical examination, assessment and skills courses and suicide prevention training. The independent prescriber qualification allows pharmacists to change patient’s medication at the review and hence ensures the timely and seamless care. Although relevant to UK practice, such skills may not be relevant in other healthcare systems. Additional communication standards in the skills section were also identified, including: the use of motivational techniques, brief interventions and an understanding that sometimes no change is appropriate and ‘planting a seed’ to prepare for the future changes may be just as important.

The Scottish Government’s policy for achieving excellence in pharmaceutical care aligns closely to similar policies in North America and Australasia [1–4]. These policies focus on the utilisation of pharmacists’ expertise to allow their full integration into multidisciplinary teams to optimise the quality of prescribing and help to deliver the best therapeutic outcomes for patients and their health [1–4]. The need for advanced pharmacist workforce is clear and there is the need to integrate the developed standards into consultant pharmacist curriculum in order to aid the successful implementation of integrated multidisciplinary working to meet local, national and international visions [1–4, 49–51].

North American and UK studies indicate that there are several factors affecting pharmacist’s abilities to take on new responsibilities and roles in multidisciplinary teams [52–57]. In part this is due to a lack of clarity of role, gaps in peer, management and support structures that inhibit optimal multidisciplinary integration, as well as some medical colleagues being anxious about pharmacists’ training and abilities [6, 57]. However, recent evidence demonstrates that the appropriate use of competency frameworks can improve the pharmacist’s performance [58]. Thus, the production of GPCP standards may provide the first step needed to further maximise the performance and effectiveness of the pharmacist clinics, by ensuring uniformity and consistency in delivery.

### Further research

Further work will be required to engage key stakeholders from primary care providers, policy makers, educational bodies to members of general practice multidisciplinary teams in order to ensure alignment and maintenance of standards within general practice. Additionally, we need to establish optimal implementation processes for these and other standards by assessing and providing the environmental and professional conditions necessary for evidencing the standards and linkage to nationally approved competency frameworks for advanced pharmacist practice [29, 32]. Furthermore, research is required to explore how to standardise the time resource needed for these reviews, as no consensus was reached on this. Finally, policy makers and professional bodies may find it of use to identify and consider core and specialists standards applicable to generalists and specialists areas of practice.

## Conclusion

This study identified the standards of practice for polypharmacy and chronic disease medication reviews in general practice. The identified standards covered seven categories-skills, environment, qualifications, qualities and behaviours, knowledge, process and experience. Similarly, to other standards for pharmacists within the UK and internationally, they concentrated on good communication and patient centeredness with ability to manage complex patients, leadership, team work as well as the ability to work independently. Some new standards, specific to polypharmacy and chronic disease medication reviews in general practice were also identified.

The production of GPCP standards may help to maximise the performance and effectiveness of the pharmacy service, as well as ensuring uniformity and consistency of service delivered. Further research will be required to engage key stakeholders in order to ensure alignment and maintenance of standards within general practice.

## Supplementary Information

Below is the link to the electronic supplementary material.

Supplementary file1 (DOCX 22 kb)
