# Supplementary material for: The standards of practice for delivery of polypharmacy and chronic disease medication reviews by general practice clinical pharmacists: a consensus study
Source: Int J Clin Pharm. 2022 Mar 23;44(3):663–72. doi: 10.1007/s11096-022-01387-7 (PMC8941365; doi:10.1007/s11096-022-01387-7)
Supplement: Supplementary file 1 — Supplementary file1 (DOCX 22 kb) [file 11096_2022_1387_MOESM1_ESM.docx]

**Supplementary File 1 – Recommendation for the Conducting and Reporting of Delphi Studies (CREDES)**

| Section/Topic | # | Where reported (page number) |
| --- | --- | --- |
| Rationale for the choice of the Delphi technique |  |  |
| *Justification.* | 1 | 9 |
| Planning and design |  |  |
| *Planning and process.* | 2 | 9 |
| *Definition of consensus.* | 3 | 9 |
| Study conduct |  |  |
| *Informational input.* | 4 | 9 |
| *Prevention of bias.* | 5 | 9 |
| *Interpretation and processing of results.* | 6 |  |
| *External validation.* | 7 | N/A |
| Reporting |  |  |
| *Purpose and rationale.* | 8 | 9 |
| *Expert panel.* | 9 | 9 |
| *Description of the methods* | 10 | 9 |
| *Procedure.* | 11 | 9 |
| *Definition and attainment of consensus.* | 12 | 9 |
| *Results.* | 13 | Table 2., p11-12, 24-25 |
| *Discussion of limitations.* | 14 | 13 |
| *Adequacy of conclusions.* | 15 | 14-15 |
| *Publication and dissemination.* | 16 | 14-15 |

**Supplementary File 2.**

Table 2. Rejected standards by category, consensus not achieved during ranking rounds of NGT (n=25).

|  | **Rejected** |  |
| --- | --- | --- |
| **Time** |  |  |
| 1. Appointment is 20 minutes | No consensus | |
| 1. Follow up issues 10 minutes. | ◊ | |
| 1. 30 min average for face to face consultation | ◊ | |
| 1. Follow up - would mean additional time - could mean 2nd consultation of phone review. 'Complex' patient, so some may take longer e.g. especially for pain, mental health, polypharmacy. | ◊ | |
| 1. Use time efficiently – documentation, avoid unnecessary documentation. | ◊ | |
| 1. Admin 15mins (1st review), 5mins (subsequent review) - pre clinic | ◊ | |
| 1. Post clinic - admin/letters - will depend on admin support if dictation - 5 mins. | ◊ | |
| 1. Dependent on role of clinic - polypharmacy will need longer than individual (e.g. Cardiology/respiratory). | ◊ | |
| **Governance** |  | |
| 1. Efficient but also some leeway for people who can’t navigate current system | ◊ |  |
| 1. Ensure targeting 'high priority patients' (i.e. use clinical skills for patients who needed most). | ◊ |  |
| 1. Keep the number of changes small -repeated consultations may be appropriate where multiple changes are necessary. | ◊ |  |
| 1. Clear explanation of thought process. | ◊ |  |
| 1. Clear plans for follow up of each patient. | Duplication |  |
| 1. Mentor (pharmacist IP with clinical experience in area) | ● |  |
| 1. Peer support/reflection/case learning. | ● |  |
| 1. Peer review. | ● |  |
| 1. Clinical supervision - may be most useful with a GP or specialist nurse prescriber for example ensure clinical competency on an ongoing basis. Pharmacist IP working in isolation mean this will be particularly important e.g. may be no other person within own team with same specialist interest. | ● |  |
| 1. Annual revalidation to clinical area (similar to medics) including consultation skills and clinical/therapeutic knowledge. | ● |  |
| 1. To work within their area of competency. | ● |  |
| 1. Part of practice multidisciplinary - evidence of reflection whether through case study, or Significant Event Analysis or real-life examples; must have been discussed with peers to show learning e.g. DATIX.   . | ● |  |
| **Knowledge** |  |  |
| 1. Demonstrate good clinical knowledge of common clinical conditions through achievement of advanced GPCP framework or MSc or equivalent. | ● |  |
| 1. Knowledge of structure you are working within. | ● |  |
| **Skills** |  |  |
| 1. Communication | ● |  |
| 1. Communication is essential for patient cantered care. Patient and MDT and wider (cross sector) | ● |  |
| 1. Skills Ability to work with wider team to develop role (1year/2 years) | ● |  |

**Supplementary File 3.**

**Clarification on UK only relevant standards and qualifications**

| Standard | Clarification |
| --- | --- |
| Has peer support – everyone has a mentor or appraisal additional to Knowledge Skills Framework. | Knowledge Skills Framework: **generic competency framework**that is intended to define and describe the knowledge and skills that NHS staff need to apply in their work to deliver quality services; provide a single consistent framework for staff reviews and development; and influence the pay progression of non-medical staff. |
| Has Royal Pharmaceutical Society membership is optional but may be advantageous as opens mentoring support and clinic information. | Royal Pharmaceutical Society: the body responsible for the leadership and support of the pharmacy profession (pharmacists) within England, Scotland and Wales. |
| Has completed consultation skills training (NES and video recording including feedback) | NES consultation skills training course: The aim of the consultation skills aspect of the course is:   - to enhance pharmacists' patient-centred consultation skills. - Tips for consulting by phone or video in place of face to face consultations is available at [Remote consulting.](https://learn.nes.nhs.scot/28943/coronavirus-covid-19/remote-consulting-and-recruitment) - E-learning modules available to help refine consultation skills are-clinical history taking, shared decision making, supporting health and behavioural change. (consultation skills: what good looks like, consulting with children and young people, consulting with people with mental health problems, consulting with people with dementia, consulting with people with physical disabilities, dealing with difficult discussions). - The participants are asked to record their own consultations in order to get comments on them as part of the module completion. |
| Has completed NES clinical examinations course (and advanced if relevant to clinical area). | NES clinical examinations course: Clinical skills for pharmacists:  **Learning Outcomes** Part 1: Demonstrate the general examination of a patient in simulation; Demonstrate safe and accurate measurement and recording of vital signs in simulation, including manual blood pressure measurement, temperature, respiratory rate, heart rate and pulse, oxygen saturations; Gain an understanding of the value and requirement for measuring lying and standing blood pressure; Demonstrate safe practice of measuring postural blood pressure in simulation; Demonstrate correct use of a stethoscope; Examine a chest in the context of a respiratory system problem; Describe assessment tools used to assist the assessment of patients (NEWS, sepsis toolkit, Assign-score)  **Learning Outcomes:** Part 2:  Demonstrate capillary blood glucose measurement; Demonstrate a basic eye assessment using the VIPEM structure (VIPEM: V - visual acuity, I - Inspection, P - Pupillary responses, EM - Eye Movements); Demonstrate a basic examination of the ears; Demonstrate a basic examination of the throat; Demonstrate a basic skin examination |
| Has completed NES communication course. | NES communication course: Basic clinical consultation skills:  **Learning Outcomes:** Apply the Calgary-Cambridge model to clinical consultations; Critique the quality of consultations using relevant criteria; Demonstrate a structured approach to the handing over of clinical information relevant to a patient's history; Describe a structured approach to a follow-up consultation |

**Supplementary File 4.**

**The pre-recorded form used for silent generation in NGT.**

| Name |  |
| --- | --- |
| Age |  |
| Post grad qualifications (please specify) | IP /Msc/Diploma/Certificate/PhD |
| Area of interest& current role |  |
| Grade AfC |  |
| Duration in the current role |  |

**The proposed standards:**

Skills

Competencies

Governance

Timing
